# Cells to the Rescue: Emerging Cell-Based Treatment Approaches for NMOSD and MOGAD

**DOI:** 10.3390/ijms22157925

**Published:** 2021-07-25

**Authors:** Judith Derdelinckx, Tatjana Reynders, Inez Wens, Nathalie Cools, Barbara Willekens

**Affiliations:** 1 Department of Neurology, Antwerp University Hospital, 2650 Edegem, Belgium; judith.derdelinckx@uza.be (J.D.); tatjana.reynders@uza.be (T.R.); 2 Laboratory of Experimental Hematology, Vaccine & Infectious Disease Institute (VAXINFECTIO), Faculty of Medicine and Health Sciences, University of Antwerp, 2610 Wilrijk, Belgium; inez.wens@uza.be (I.W.); nathalie.cools@uza.be (N.C.); 3 Translational Neurosciences, Faculty of Medicine and Health Sciences, University of Antwerp, 2610 Wilrijk, Belgium; 4 Center for Cell Therapy and Regenerative Medicine, Antwerp University Hospital, 2650 Edegem, Belgium

**Keywords:** NMOSD, neuromyelitis optica spectrum disorders, MOGAD, MOG antibody-associated disease, cell therapy, dendritic cell, CAR-T cell, mesenchymal stem cell, hematopoietic stem cell transplantation, tolerance, immunotherapy

## Abstract

Cell-based therapies are gaining momentum as promising treatments for rare neurological autoimmune diseases, including neuromyelitis optica spectrum disorders and myelin oligodendrocyte glycoprotein antibody-associated disease. The development of targeted cell therapies is hampered by the lack of adequate animal models that mirror the human disease. Most cell-based treatments, including HSCT, CAR-T cell, tolerogenic dendritic cell and mesenchymal stem cell treatment have entered early stage clinical trials or have been used as rescue treatment in treatment-refractory cases. The development of antigen-specific cell-based immunotherapies for autoimmune diseases is slowed down by the rarity of the diseases, the lack of surrogate outcomes and biomarkers that are able to predict long-term outcomes and/or therapy effectiveness as well as challenges in the manufacturing of cellular products. These challenges are likely to be overcome by future research.

## 1. Introduction

Various types of cell-based therapies may hold promise for treatment of potentially severe autoimmune neurological diseases, including neuromyelitis optica spectrum disorders (NMOSD) and myelin oligodendrocyte glycoprotein antibody-associated disease (MOGAD). Neuromyelitis optica, or NMO, was first described by Eugène Devic in the late 19th century as a variant of multiple sclerosis (MS), presenting with optic neuritis and myelitis [1]. In 2004, antibodies towards aquaporin-4 (AQP4) were discovered and NMO could clearly be distinguished from MS [2,3]. The term NMOSD was introduced for the first time in 2007 [4]. It was recognised that not only presentations with optic neuritis and/or (longitudinally extensive or short) transverse myelitis could occur. Indeed, a spectrum of clinical presentations including area postrema, diencephalic, brainstem and symptomatic cerebral syndrome has been associated with AQP4-IgG antibodies, leading to renaming of NMO to NMOSD [4,5]. However, in some NMO patients, AQP4-IgG antibodies could not be detected and this category was named ‘seronegative NMO’. Later, it became clear that a proportion of these seronegative NMO patients carried autoantibodies towards another autoantigen, myelin oligodendrocyte glycoprotein (MOG). This disease is now referred to as MOG antibody-associated disease or MOGAD [6]. Clinical presentations include optic neuritis, myelitis, brainstem syndromes, acute disseminated encephalomyelitis (ADEM), but the disease spectrum is expanding with rare clinical presentations such as (but not limited to) unilateral cortical encephalitis [7]. Today, MOGAD is increasingly regarded as a separate disease entity from AQP4-IgG positive (AQP4+) NMOSD [8,9]. Finally, in double seronegative NMOSD, AQP4 or MOG antibodies are not demonstrable and more research is needed to better define this category of patients [10]. While AQP4+ NMOSD has been associated with other non-organ and organ-specific autoantibodies, this is less clearly the case for MOGAD [11]. However, more recently, reports of coexisting NMDAR antibodies [12] and concurrent peripheral and central demyelination with concomitant presence of anti-neurofascin antibodies [13] have challenged this concept. Both NMOSD and MOGAD diseases are accompanied by relapses and episodes of remission that are variable in duration [14].

Relapses are treated with high-dose intravenous methylprednisolone, plasma exchange or intravenous immunoglobulins (IVIg) [15]. To prevent relapses and related disability, these diseases are currently treated off-label with the anti-CD20 (cluster of differentiation) monoclonal antibody rituximab, immunosuppressants such as azathioprine, mycophenolate, methotrexate, tocilizumab—a monoclonal antibody towards interleukin (IL)-6 receptor (Il-6R)—and repeated courses of IVIg, sometimes in combination with low-dose steroids [15]. More recently, the first treatments for NMOSD have been approved by regulatory agencies, based on results of phase III randomised controlled clinical trials: satralizumab (Enspryng^®^) [16], a monoclonal antibody against the IL-6R, eculizumab (Soliris^®^) [17], an anti-C5 complement inhibitor and inebilizumab (Uplinza^®^) [18], a monoclonal antibody leading to lymphocytolysis after binding to CD-19 on B cells and plasmablasts [19]. In severe cases, autologous hematopoietic stem cell transplantation (HSCT) has been used [20]. There is no evidence-based guideline for the treatment of MOGAD, which is based on case series and expert opinion, as in this disease there have been no phase III randomised controlled trials to confirm efficacy of any of the aforementioned treatments [7].

However, chronic immunosuppressive treatments may increase the risk of infections [21] in the long term, highlighting the need for temporary and/or more targeted antigen-specific treatments, leaving protective immunity to fight pathogens and cancer intact. Cell-based therapies aim to do precisely this: either depletion of autoreactive effector cells, or modulation of autoreactive T and B cell responses, resulting in the restoration of tolerance. Regarding the latter, while some cell-based therapies target and try to modulate only the antigen-specific autoreactive T and B cells, immune reconstitution cell-based therapies, such as HSCT, are accompanied by a general and temporary severe immunosuppressive state, aiming to eradicate aberrant immune responses towards self-antigens, while restoring immunity towards non-self-antigens.

After providing an overview of the current knowledge on immunopathogenesis, the fundamental, translational and clinical research approaches in the field of cell-based therapies in NMOSD and MOGAD are reviewed and challenges and areas open to research are discussed.

## 2. Immunopathogenesis

**Figure 1:**
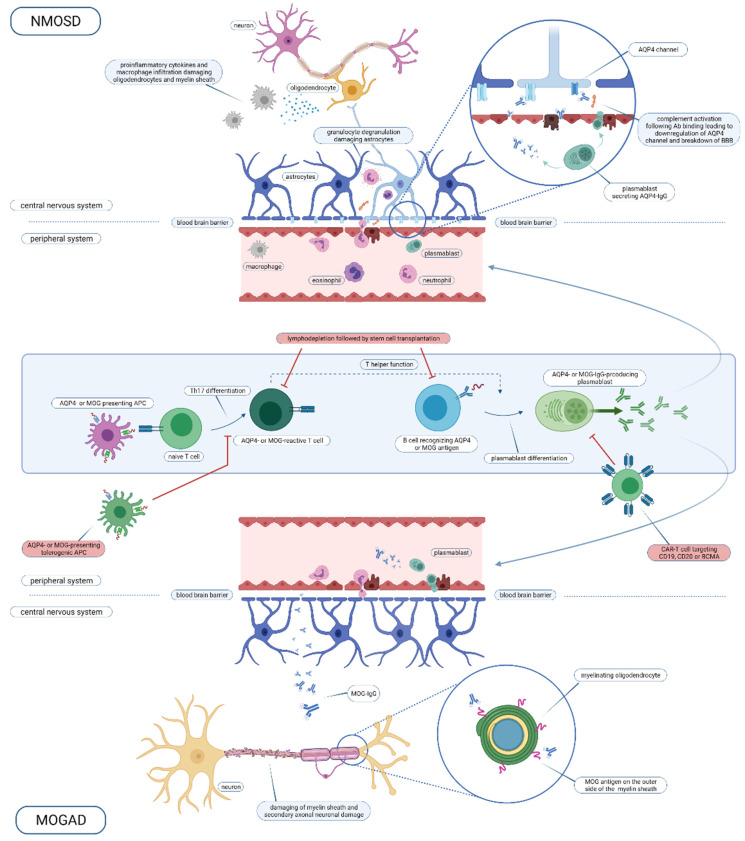
Overview of the NMOSD and MOGAD immune pathogenesis and intervention points for cell-based therapy. Abbreviations used: NMOSD, neuromyelitis optica spectrum disorder; AQP4, aquaporin-4; Ab, antibody; BBB, blood brain barrier; IgG, immunoglobuline G; MOG, myelin oligodendrocyte glycoprotein; APC, antigen-presenting cell; Th, T helper; CAR-T, chimeric antigen receptor T cell; CD, cluster of differentiation; BCMA, B cell maturation antigen; MOGAD, MOG-antibody disease. Created with BioRender.com.

Both NMOSD and MOGAD are autoimmune central nervous system (CNS) disorders, which share, in part, clinical presentations, but differ in immunopathogenesis and pathological characteristics [22]. In this section, a comprehensive insight into the pathogenesis of both diseases will be given. For a graphical overview, including intervention points for cell therapy, we refer to Figure 1.

### 2.1. AQP4 + NMOSD

In 2003, Peter Agre and Roderick MacKinnon received the Nobel prize for chemistry, honouring their work on the identification and functional characterisation of aquaporines [23]. Aquaporines are selectively permeable to water and thereby control water homeostasis. In 1994, AQP4 was first cloned from rat lung [24]. AQP4 is a cell membrane crossing water channel, which is highly expressed on astrocyte foot processes and ependymal cells in the blood–brain and blood–cerebrospinal fluid barriers in the CNS in the optic nerve, spinal cord, hypothalamus and area postrema [25,26]. However, AQP4 is also expressed in other tissues, such as kidney, stomach, airways, glands and skeletal muscle [27]. The differential expression, both within and outside the CNS, and variation in macroscopic aggregation of tetramers between tissues, are explanations why AQP4 antibodies bind to CNS predilection sites [28,29].

AQP4-IgG antibodies have been demonstrated to be pathogenic [30]. Both AQP4-IgG-producing plasmablasts and AQP4-IgG antibodies are able to cross the blood–brain barrier. Next, antibodies bind to AQP4, leading to complement-dependent cytotoxicity and chemotaxis of immune cells, including T and B lymphocytes, macrophages, neutrophils and eosinophils. Neutrophils degranulate and lead to astrocyte death, in its turn causing oligodendrocyte death. Hence, the severe inflammatory reaction leads to tissue necrosis with demyelination, axonal and neuronal damage [26]. Pathology of acute lesions shows oligodendrocyte and astrocyte loss, confluent demyelination, abundant complement deposition and a predominant CD4+ T lymphocyte inflammatory cell infiltration [22]. Besides antibodies, AQP4-specific T cells play a role in the development of NMOSD lesions. Indeed, AQP4-IgG belongs to a T cell-dependent immunoglobulin subclass (IgG1). A unifying explanation of the pathogenesis is that T cells are involved in the early onset of the disease in the periphery, leading to a breach of tolerance and subsequent antibody production by development of antigen-producing B cells. AQP4-specific T cells are sufficient to induce NMO in a mouse model, independent of antibodies [31]. Moreover, AQP4-specific T cells are amplified in patients with NMOSD versus healthy controls. Both naïve pre-germinal centre B cells (CD19^+^CD27-IgD^+^) and post-germinal centre cells (CD19^+^CD27^+^) are able to differentiate to secrete AQP4 antibodies [32]. This suggests an early, pre-germinal centre loss of immunological tolerance [32]. However, patients with AQP4+ NMOSD have higher frequency of regulatory B cells (IL-10-producing B cells, CD19^+^CD39^+^CD1d^+^IL-10^+^) [33,34]. Functional properties of B cells have not been investigated yet. Besides an important role of the adaptive immune system, the innate immune system has also been implicated in lesion initiation. In a mouse model, it has been shown that crosstalk between astrocytes and microglia involving early-activated CNS-intrinsic complement components and microglial C3a receptor signalling is a critical driver in the evolving NMO lesion [35].

Pathologically, demyelination with preferential loss of myelin-associated glycoprotein (MAG) and oligodendrocyte loss, severe astrocytic damage and perivascular-activated complement deposits are the hallmarks of AQP4+ NMOSD [22].

The origin of the AQP4 antibodies remains elusive. Generated most likely in the peripheral immune system, these antibodies can enter the CNS following a break in the blood–brain barrier, again for a yet unresolved reason, and initiate the inflammatory cascade as described above and as depicted in Figure 1. There are several lines of evidence for their generation in the peripheral immune system: (1) AQP4 antibodies have been demonstrated in the serum long before the development of NMOSD [36]), (2) in contrast to MS, oligoclonal bands reflecting intrathecal IgG synthesis are mostly absent in NMOSD [37] and (3) predilection sites are the area postrema and the posterior pituitary, which are not covered by the blood–brain barrier—hence first presenting symptoms consistent with a lesion location in this areas may be regarded as a sign of relocation of AQP4 antibodies from the peripheral system to the brain (as reviewed by [26]). AQP4+ NMOSD has been described as a paraneoplastic disorder [38], which suggests that mechanisms of molecular mimicry with tumour antigens may play a role in the development of the disease. The role of molecular mimicry has also been suggested by the fact that AQP4-specific T cells display cross reactivity towards a bacterial protein (*Clostridium perfringens* adenosine triphosphate-binding cassette (ABC) transporter permease) [39]. Another *Clostridium* species (*C. bolteae*) has recently been implicated in the pathogenesis of NMOSD in Indian patients [40]. Environmental risk factors, especially herpes virus infections, are not clearly associated with NMOSD, unlike MS [41]. *HLA* associations have been described in Japanese (HLA-DRB1*08:02 and HLA-DRB1*16:02) and Dutch populations (HLA-A*01, -B*08 and -DRB1*03) [42,43,44,45].

While major advances in the knowledge of NMOSD pathophysiology have led to investigating the efficacy of targeted treatments in phase III clinical trials, further elucidation of the immunopathogenesis of AQP4+ NMOSD may lead to novel treatment approaches. Besides IL-6, CCL22 and CCL3, CD16^+^CD56^+^ NK cells and CX3CL1 have been identified as potential novel biomarker candidates [46]. Besides the assessment of the frequency of various immune cell types [46], their functional characteristics need further exploration. For instance, the role and function of antigen-presenting cells, such as dendritic cells, have not been clarified so far. Moreover, seronegative NMOSD requires further research.

### 2.2. MOGAD

Recently, the immunopathology of 11 patients who underwent brain biopsy, and another 24 patients who underwent brain biopsy or for whom autopsy material was available, was described extensively [22,47]. Within the lesions, perivenous demyelination with inflammatory infiltrates consisting of mononuclear cells and/or macrophages, but fewer polymorphonuclear cells, was seen. Axons were relatively preserved and slowly expanding lesions were not present, in contrast to MS pathology [47]. Most lesions showed MOG-predominant myelin loss while other myelin proteins were spared. Myelin phagocytic macrophages were present not only in the lesions but also in the perivascular spaces. The perivascular cellular infiltrates consisted mainly of CD4+ T cells and a lower number of CD8+ T cells and B cells [22]. This is clearly different from MS lesions, where mainly CD8+ T cell infiltrates are found. Only in one MOGAD patient was activated complement seen. In seven patients, IgG staining was seen in the perivenous demyelinated lesions. In the cortico-medullary junction, demyelination and T cell infiltration were also the dominant processes, while axons and oligodendrocytes were relatively preserved [22]. Intracortical demyelination was overrepresented in comparison to classical MS [47]. These pathological observations have to be contrasted with the fact that, to date, no MOG-specific T cells have been found in the peripheral blood of patients with MOGAD [48]. This could be explained by the fact that these MOG-specific T cells are difficult to detect due to their low frequencies. However, this remains to be demonstrated in research. On the other hand, MOG-specific B cells have been detected in samples of patients [25,49]. MOG antibodies seem to have a direct role in the pathogenesis of the disease as suggested by animal models, but cotransfer with myelin-reactive T cells is needed [50,51].

Genetic risk factors for MOGAD remain largely unknown. While an *HLA* association has been described in NMOSD, no *HLA* associations have been found in a UK [52] and a Dutch population of MOGAD patients [44]. Only in a Chinese cohort of patients with paediatric-onset MOGAD was an association with HLA-DQB1*05:02-DRB1*16:02 alleles found. MOGAD has been described as occurring after infections [53,54] or more recently as a paraneoplastic syndrome in association with an ovarian teratoma [55]. However, these are only case reports and systematic research on environmental risk factors for MOGAD is lacking. A lack of seasonal variation in MOGAD attacks may argue against a significant role for environmental factors [56].

## 3. Experimental Animal Models of NMOSD and MOGAD

### 3.1. Animal Models for NMOSD

As recently reviewed by Duan et al., a multitude of animal models for AQP4+ NMOSD have been developed over the past two decades [57]. These models all partially resemble clinical and pathological features of human NMOSD, which is characterised by spontaneous development of CNS inflammation in a relapsing manner, predominantly targeting spinal cord and optic nerves with relative cerebral sparing. Most of these models are based on human AQP4 IgG administration, with or without pro-inflammatory interventions, or on passive transfer of AQP4-reactive T cells. The earliest models originated from administration of AQP4 IgG in animals with experimental autoimmune encephalomyelitis (EAE), one of the animal models for MS, in which transfer of AQP4 IgG to the CNS was facilitated by breakdown of the blood–brain barrier in the context of EAE. Other NMO-specific animal models were developed later on by administration of AQP4 IgG either directly into the CNS (brain parenchyma or spinal cord) of by systemic administration, both intravenously or intraperitoneally, in combination with manoeuvres to damage the blood–brain barrier, e.g., targeted ultrasound. Finally, passive transfer of AQP4-reactive T cells in rodents induced spinal cord and optic nerve inflammation, albeit without AQP4 loss [58].

Additionally, EAE animal models with NMO resemblance by the specific occurrence of optic neuritis and myelitis have been described without the use of AQP4 IgG or AQP4-reactive T cells for disease induction. For example, opticospinal models of demyelination have been generated in Brown Norway and Lewis rats by administration of recombinant MOG in incomplete Freund’s adjuvant [59,60,61,62]. Apart from the clinical NMO phenotype, some NMO-like pathological features were demonstrated, including astrocyte apoptosis [62]. However, no AQP4 antibodies could be detected, and furthermore, involvement of the periventricular regions was visualised on brain MRI, which are both atypical findings for NMO. Although originally presented as animal models for NMO in terms of their clinical phenotype, these full-length MOG-induced demyelinating models appear to show more resemblance to MOGAD, as discussed further on.

Finally, recently, a Lewis rat model, using mimotopes (peptides, which mimic the conformational AQP4 epitopes), was described as a model to study tolerance induction [63].

Historically, all of these NMOSD animal models have mainly contributed to our understanding of NMO disease pathogenesis. For example, the pathogenicity of AQP4 IgG, as well as the role of complement and antibody-dependent cell cytotoxicity in NMO pathogenesis, has been demonstrated by means of animal models. However, animal models were not involved in recent major therapeutic breakthroughs for NMO, such as eculizumab [17,64]. This is driven by some major limitations of the currently available NMOSD animal models [57], including the lack of animal models with the development of spontaneous AQP4-directed autoimmunity, the intrinsic bias of these animal models towards the cellular or humoral compartment and the fact that most NMOSD animal models are murine, which is problematic due to the fact that mice do not have a functional complement pathway [65]. Finally, no animal model representing seronegative NMO is available up to the present date. Hence, there is a need for more representative NMOSD animal models, in order to facilitate clinical translation.

### 3.2. Animal Models for MOGAD

To our knowledge, no specific animal model for MOGAD has been developed so far. However, some animal models for MS closely resemble MOGAD pathology, including the full-length MOG-induced opticospinal demyelination in rats described earlier on, as well as the 2D2 EAE model, in which MOG T cell receptor (TCR) transgenic mice spontaneously develop severe optic neuritis [66]. Moreover, when crossed with IgHMOG mice, in which a significant proportion of B cells are MOG-reactive, a transgenic model carrying both MOG-specific B and T cell arises, leading to the spontaneous development of severe myelitis and optic neuritis with relative sparing of the brain [67], similar to MOGAD. A cynomolgus macaque model of EAE, in which administration of recombinant human MOG (rhMOG) elicits brain inflammation mediated by MOG-autoreactive CD4+ lymphocytes and anti-MOG IgG, also mimics the immunopathology of MOGAD [51]. In this model, a recombinant antibody directed against the dendritic cell-asialoglycoprotein receptor (DC-ASGPR) fused to MOG, led to induction of MOG-specific CD4+CD25+FOXP3+CD39+ regulatory lymphocytes and protection from developing EAE [51].

## 4. Cell-Based Therapies

Various cellular treatment approaches have been investigated in NMOSD and occasionally in MOGAD as well. These have been used either in the controlled setting of clinical trials, or as a rescue therapy for highly aggressive disease in individual patients. The results of these clinical trials and case reports are discussed below. For an overview of registered, completed, ongoing and withdrawn clinical trials in this field, we refer to Table 1.

### 4.1. Tolerance-Inducing Dendritic Cells

One phase Ib, open-label, multiple ascending dose, single-centre clinical trial has investigated the safety and feasibility of intravenously administered autologous tolerogenic peptide-loaded dendritic cells (DC) in four AQP4+ NMOSD patients in Spain [68]. Here, the tolerogenic phenotype of DC was induced by addition of dexamethasone. DC from NMOSD patients were stimulated with seven myelin peptides (MBP_13–32_, MBP_83–99_, MBP_11–129_, MBP_146–170_, MOG_1–20_, MOG_35–55_, PLP_139–154_) and AQP4_63–76_, which was previously shown to be immunogenic in vitro [39]. Three doses of tolerogenic DC were administered intravenously at week 0, 2 and 4 at progressively increasing doses, including 50 × 10^6^, 100 × 10^6^, 150 × 10^6^ and 300 × 10^6^ DC. Following treatment, the patients entered a safety follow-up phase in which they were followed up to 24 weeks. All NMOSD patients received concomitant treatment with rituximab (3) or mycophenolate (1). One NMOSD patient had elective surgery, not related to the experimental treatment, and this event was classified as a serious adverse event (SAE). All patients remained clinically stable and no relapses occurred. Two patients experienced four adverse events, including back pain, left leg pain, influenza and palpitations. Immunological evaluations demonstrated a trend for decreased T cell proliferation as measured by [^3^H]-thymidine incorporation in response to AQP4 peptide at week 12 as compared to baseline [68]. A significant increase of IL-10 (interleukin-10) production, measured with ELISA in response to peptide stimulation in PBMC culture supernatant, was demonstrated at week 12 compared to baseline for AQP4 [68]. This was accompanied by an upward trend in the frequency of type 1 regulatory T (Tr1) cells. In conclusion, this tolDC-based therapy was safe in NMOSD patients and immunological analysis demonstrated changes compatible with tolerance induction.

### 4.2. Hematopoietic Stem Cell Transplantation in NMOSD and MOGAD

In hematopoietic stem cell transplantation for autoimmune diseases, the aim is to destroy the aberrantly functioning immune system with high-dose chemotherapy and rebuild it by hematopoietic stem cell infusion, thereby aiming to induce long-term disease remission. The therapeutic potential lies in aggressive immunosuppression while the HSC are needed for recovery of the immune system. In patients with autoimmune diseases, autologous transplantations have been preferred above allogeneic ones to prevent graft-versus-host reactions and related morbidity and mortality. A recent meta-analysis included three studies (published between 2000 and 2020) on 31 NMOSD patients who underwent AHSCT [69,72,73,74]. The progression-free survival (PFS = survival without progression; progression = worsening of neurologic disability beyond the pre-treatment baseline (increase in EDSS > 1 with a pre-transplant baseline EDSS score of ≤5 or >0.5 with a baseline EDSS score of >5)) was 76% during a follow-up period between 2 and 13 years. Treatment-related mortality (TRM = death within 100 days of AHSCT) was 0% [72]. No results on immunomonitoring of peripheral blood immune cell subsets were available. Guidelines from the European Bone Marrow Transplantation (EBMT) Autoimmune Diseases Working Party (ADWP) recommend the use of AHSCT in NMOSD as a clinical option, with grade II evidence, in therapy-refractory patients [75]. Despite the promising results, a number of patients had persisting AQP4 antibodies and relapsed within 5 years [69,73]. Moreover, the optimal conditioning regimen remains unclear to date.

Only a few cases of NMOSD patients who underwent allogeneic HSCT (alloHSCT) were reported (summarised in [20,76,77,78]). AlloHSCT has the potential to clear all autoreactive lymphocytes by allogeneic donor T lymphocytes (graft versus autoimmunity), thereby leading to a more profound immunotherapeutic effect. However, this needs to be balanced with the more significant risks of morbidity and mortality after alloHSCT. Several immune-mediated peripheral and central nervous system diseases, including a case of MOGAD, have been reported after alloHSCT in haematological patients, urging for prudence when using alloHSCT in patients with autoimmune diseases [79]. Due to limited clinical evidence, alloHSCT in NMO was classified as developmental by the EBMT-ADWP [75] and is currently not recommended as a clinical option.

Only one reported case of a 25-year-old treatment-refractory male with MOGAD was found in the literature, who recovered very well after AHSCT [80]. Follow-up duration was less than one year after AHSCT, while his disease started as early as the age of 10 years [80].

In conclusion, the evidence for HSCT in NMOSD and MOGAD is limited and a proportion of patients will relapse within 5 years. AHSCT can be considered as a clinical option to treat treatment-refractory NMOSD patients according to EBMT [75]. Concerning the use of (allo)HSCT in NMOSD and MOGAD with (allo)HSCT, more research in this field is necessary to determine short- and longterm safety, efficacy and optimal condition regimens.

### 4.3. CAR-T Cell Therapy

Chimeric antigen receptors (CAR) are receptor proteins carrying both an antigen-binding and a T cell-activating function, allowing T cells to target a specific protein. CARs consist of an extracellular binding domain containing the single-chain variable fragment from an antigen-reactive antibody, allowing antigen-specific binding, and an intracellular domain containing the CD3ζ chain domain, allowing T cell receptor signalling and T cell activation after antigen binding to the extracellular domain [81]. Following in vitro genetic modification of T lymphocytes, mostly using viral transduction but more recently also using the CRISPR/Cas9 technique [82], T cells expressing such CAR proteins can be readministered to a patient, where they induce a protein-specific immune response. CAR-T cell therapy was originally developed in the field of cancer therapy, where it was designed to target specific proteins expressed on tumour cells, inducing a tumour-specific cytotoxic response. Most success has been achieved in the treatment of haematological cancers, including lymphoma and leukaemia [83]. Treatment of solid tumours using CAR-T cell therapy has appeared to be more challenging, mainly due to the lack of identification of tumour-specific antigens and challenges in terms of infiltration and survival of CAR-T cells within the solid tumour microenvironment [84].

B cell targeting using CAR-T cell therapy focuses on B cell markers CD19, CD20 and B cell maturation antigen (BCMA) as antigenic targets, for instance, in the field of B cell leukaemia [85], B cell lymphoma [86,87] and multiple myeloma [88]. Next to the oncological field, B cell overactivation has also been implicated in the field of autoimmunity, where dysregulated B cell activation leads to an antibody-mediated targeting of healthy own body tissue. Hence, B cell targeting using CAR-T cell therapy shows additional promise for the treatment of autoimmune diseases. Breaking the immune tolerance towards autoreactive immune cells induces specific cytotoxic death of these cells, which may downregulate the immune overactivation driving autoimmunity. Indeed, CAR-T cell therapy targeting CD19 has recently been demonstrated to be effective in the prevention and treatment of a murine model of systemic lupus erythematosus [89].

In the field of NMOSD, a first clinical trial evaluating the safety and efficacy of CD19 and CD20 CAR-T cell therapy was withdrawn due to recruitment difficulties (ClinicalTrials.gov NCT03605238). Currently, an open-label phase I clinical trial is ongoing, using BCMA CAR-T cell therapy in patients with refractory AQP4-IgG-seropositive NMOSD (ClinicalTrials.gov NCT04561557). Twelve NMOSD patients will be enrolled, receiving BCMA CAR-T cells following lymphodepletion with cyclophosphamide and fludarabine. Primary outcome measures include the incidence of dose-limiting toxicities and adverse events. The concentration of AQP4-IgG titers in the serum 3 months after infusion and the CAR-T cell proliferation 2 years after infusion will be studied as secondary outcome measures, together with clinical and radiological outcome measures, including annualised relapse rate and active MRI lesions. The first results of this clinical trial are expected by the end of 2023.

In line with the development of CAR-T cells for targeted cytotoxic depletion of autoreactive T cells by breaking of tolerance, another approach is the generation of antigen-specific regulatory T cells (Treg) by transfection with TCRs specific for particular auto-antigens, aiming at antigen-specific tolerance induction. Indeed, in addition to previous clinical trials demonstrating safety and efficacy of polyclonal Treg administration for the treatment of graft-versus-host disease [90,91,92] and type 1 diabetes mellitus [93,94], next-generation antigen-specific CAR-Treg therapies are being developed, albeit still in the preclinical stage (as reviewed by [95]). Although, to our knowledge, no such translational research is currently being performed in the field of NMOSD or MOGAD, this may form an interesting research avenue.

### 4.4. Mesenchymal Stem Cell Transplantation

Mesenchymal stem cells (MSC) are multipotent stromal progenitor cells, derived from allogeneic umbilical cord tissue, autologous bone marrow or autologous adipose tissue. Although the therapeutic effect of MSC treatment was historically presumed to be driven by the regeneration of damaged tissue, additional beneficial effects have been demonstrated, including an immunomodulatory action by inhibition of the release of pro-inflammatory cytokines from both innate and adaptive immune cells and a neuroprotective action by secretion of neurotrophic and survival-promoting growth factors [96,97,98,99,100]. In the field of NMOSD, clinical trials with both bone marrow-derived MSC (b-MSC) [70] and umbilical cord tissue-derived MSC (hUC-MSC) [71,101] have been conducted.

A pilot study evaluating safety and feasibility of a single intravenous infusion with autologous bMSC in 15 AQP4 IgG+ NMO patients was completed in 2016 [70]. Previous immunomodulatory treatment (cyclophosphamide, azathioprine, with or without corticosteroids) was stopped 30 days before bone marrow harvest and patients were followed for 24 months after the bMSC infusion. No adverse events were observed. At 12 months, the mean annualised relapse rate (ARR) was reduced in comparison to pre-treatment ARR (1.1 versus 0.3, *p* = 0.002), accompanied by a decrease in T2 or gadolinium-enhancing T1 lesions in the optic nerve and the spinal cord on MR imaging. At 24 months, 13 patients (87%) were still relapse-free and disability had improved in 6 patients (40%), providing encouraging indications of a beneficial clinical effect of bMSC treatment in NMO. Due to lack of tracking experiments or pathological data, the exact mode of action of bMSC transplantation, however, remained unclear. It was postulated that the beneficial effect of bMSC transplantation was driven by both immune response modulation and promotion of tissue recovery and repair, based upon findings of (1) a decrease in T follicular helper cell counts and accompanied attenuation of pro-inflammatory IL-6 and IL-21 cytokine levels and (2) a significant thickening of the retinal nerve fibre layer, an increase in the optic nerve diameter and an enlargement of the upper cervical area [70].

In contrast to bMSC, hUC-MSC are easily collectable and, although not autologous, these MSC have a low risk of induction of allogeneic immune responses and hence transplant rejection [102]. In 2012, a phase I clinical trial evaluated the effect of treatment with hUC-MSC in five AQP4 IgG+ NMO patients [101]. The cells were administered by intravenous and intrathecal route combined, divided over four infusions. Average number of relapses decreased significantly following transplantation, compared to pre-treatment relapse rate (1.4 versus 3.2, *p* < 0.05). EDSS score improved in four out of five patients 24 months following transplantation. In these four patients, peripheral blood B lymphocyte fraction decreased compared to pre-treatment, although the exact meaning of this finding remains elusive [101]. Recently, the results from the 10-year follow-up of these patients have been published [71]. Importantly, no long-term adverse events were detected, in particular no tumour formation or peripheral organ disorders. In the extended follow-up period, four out of five treated NMO patients demonstrated reduced annual relapse occurrence compared to before treatment. However, only two patients completed the 10-year follow-up period, due to death in two patients and loss-to-follow-up of one patient. The authors stated that these deaths were caused by rapid disease progression and were not a direct consequence of the transplantation [71]. In conclusion, long-term clinical evidence following hUC-MSC transplantation remains scarce and further clinical trials are warranted.

## 5. Discussion

Autoimmune diseases with well-known target antigens, such as AQP4+ NMOSD and MOGAD, are good candidate diseases to investigate tolerance-inducing cell-based therapies, especially in an antigen-specific way. While next-generation cell-based therapies have entered the arena of cancer treatment [103], they are still in their infancy in the field of autoimmune diseases. Several challenges need to be solved to drive the field of tolerance-inducing cell-based therapies in NMOSD and MOGAD forward.

One major challenge is the lack of adequate experimental animal models that mimic the human disease in such a way that these models can be used for translational research. Hence, it is difficult to obtain preclinical data as incentive towards clinical translation. However, progress is being made in this field.

As both NMOSD and MOGAD are rare diseases, multicentre clinical trials are necessary to achieve sufficient power to detect clinical efficacy. Recent world-wide phase III randomised placebo-controlled clinical trials, investigating the efficacy of satralizumab (Enspryng^®^) [16], eculizumab (Soliris^®^) [17] and inebilizumab (Uplinza^®^) [18] have led to the registration of the first evidence-based treatments for AQP4+ NMOSD. Hence, in the near future, it will become increasingly difficult to design and execute clinical trials investigating tolerance-inducing cell-based therapies in this field. No phase III multicentre randomised clinical trials have been performed to assess treatment effectiveness in MOGAD. Due to the low prevalence of this disease, the wide age range and broad clinical spectrum, it will be a major challenge to set up and conduct a large, multicentre, randomised clinical trial [7].

A reliable biomarker to predict future relapses or disability is not available to date. This makes proof-of-concept clinical trial design, in which biomarkers are important to detect a hint of effectiveness, challenging and prone to selection of very active patients with high relapse activity. Conflicting evidence exists on the clinical usefulness of antibody titers in the follow-up of patients with MOGAD or NMOSD. Antibody titers of AQP4 antibodies [104,105,106] are not predictive of future relapses. MOG antibodies [107,108] are higher during relapse than in remission [109] and conversion to antibody-negative status has been correlated with a higher chance of having a monophasic disease course [110,111]. In children, persisting MOG antibodies have been correlated with a higher risk of relapse [107]. However, this is not absolute: patients can remain clinically stable with positive MOG antibodies for months to years [112,113]. Hence, 6-monthly retesting of MOG antibodies, for up to 2 years or until antibodies become negative, has been recommended by the European Paediatric MOG Consortium [114]. Intrathecal production of MOG antibodies occurs more frequently than that of AQP4 antibodies [115]. The diagnostic and prognostic value of CSF unique MOG antibodies deserves more research [116]. Recently, serum glial fibrillary acidic protein (sGFAP) has gained attention as a potential biomarker to assess disease severity and future disease activity in patients with AQP4+ NMOSD in remission [117]. Biomarkers in MOGAD are the subject of ongoing research [114].

Moreover, it is not clear if cell-based therapy would be sufficient as monotherapy or if combination or adjuvant treatment protocols should be aimed for. Even HSCT is not able to induce long-term remission as many NMOSD patients will continue to relapse (in one study, there was relapse-free survival of 31% and 10% after 3 and 5 years in 16 patients) and they may need other treatments [73]. One strategy of theoretical interest is starting with a strong immunosuppressive treatment, aiming to eradicate disease-causing lymphocytes, followed by a maintenance treatment to maintain immune tolerance over the long term. This concept has been applied in the treatment of MS, albeit with variable results [118,119,120]. As antigen-specific T cells and antibodies are both involved in lesion formation in NMOSD, it is likely that T cell responses should be modulated, but pathogenic antibodies should be cleared as well. In MOGAD, MOG-specific T cells have not been detected in the peripheral blood of patients to date [48]. In this study, antigen-specific T cell responses were measured with carboxyfluorescein diacetate succinimidyl ester proliferation assay and the detection of granulocyte macrophage colony-stimulating factor (GM-CSF), interferon (IFN)-ɤ and IL-4, IL-6 and IL-17A in cell culture supernatants. Nine MOG-peptides were used to stimulate antigen-specific T cells [48]. This result contrasts with the pathological findings, in which CD4+ T cells are abundantly present. Single-cell technology to detect rare immune cell populations or analysing T cells in CSF samples may provide some answers here.

Finally, the manufacturing process of autologous cell-based therapies, such as tolDC and CAR-T cells, is labour intensive and the logistic process from apheresis to administration of the final cell product is complex. On-site manufacturing could be a solution for this; however, harmonization of culture protocols over different study sites may be an issue.

Moreover, it is not clear to date if progenitor cells from patients with NMOSD or MOGAD are of the same quality as healthy controls, e.g., for bMSC (decreased proliferation rate, more prone to senescence) [121], and optimization of culture protocols may be warranted.

## 6. Conclusions

Cell-based therapies are in the developmental stage for rare neuroinflammatory diseases, such as AQP4+, NMOSD and MOGAD. Numerous challenges in the development and clinical translation lie ahead, which are likely to be overcome by future research. 

## Figures and Tables

**Table 1:** Overview of completed, ongoing and withdrawn clinical trials with cell therapy in the field of NMOSD and MOGAD, as registered in the ClinicalTrials.gov database. Abbreviations used: MOGAD, MOG antibody-associated disease; AQP4, aquaporine 4; a.o., amongst others; N/A, not applicable; MS, multiple sclerosis; NMOSD, neuromyelitis optica spectrum disorder; CAR, chimeric antigen receptor; CD, cluster of differentiation; EDSS, Expanded Disability Status Scale.

Title	ClinicalTrials.gov Identifier	Status	Type of Trial	Intervention	Study Population	Primary Outcome	Results	Reference
**Dendritic cells**								
Treatment of Multiple Sclerosis and Neuromyelitis Optica With Regulatory Dendritic Cell: Clinical Trial Phase 1 B	NCT02283671	Completed	Dose-escalating phase I	Tolerogenic dendritic cells loaded with myelin and AQP4 peptides	MS and NMOSD	Adverse events	Well tolerated, without serious adverse events and with no therapy-related reactions	Zubizaretta et al. [68]
**Hematopoeitic stem cells**								
Hematopoietic Stem Cell Transplant in Devic’s Disease	NCT00787722	Completed	Phase I/II	Hematopoeitic stem cells, after preconditioning with a.o. cyclofosfamide and rituximab	NMOSD	Survival	11/13 patients survived more than 5 years post-transplant	Burt et al. [69]
Autologous Hematopoietic Stem Cell Transplant in Neuromyelitis Optica (SCT-NMO)	NCT01339455	Terminated (recruitment failure)	Phase I/II	Autologous hematopoeitic stem cells, after preconditioning with a.o. cyclofosfamide and rituximab	NMOSD	Proportion relapse-free at three years	N/A	N/A
Autologous Transplant To End NMO Spectrum Disorder	NCT03829566	Withdrawn by investigator	Open-label phase II/III	Autologous hematopoietic stem cells, after preconditioning with a.o. cyclofosfamide and rituximab	NMOSD	Progression-free Survival	N/A	N/A
Autologous Peripheral Blood Stem Cell Transplant for Neurologic Autoimmune Diseases	NCT00716066	Recruiting	Open-label phase II	Syngeneic or autologous hematopoietic stem cells, after high-dose preconditioning regimen with high-dose carmustine, etoposide, cytarabine, melphalan and antithymocyte globulin	Severe and refractory autoimmune disorders of the central or peripheral nervous system (including NMOSD)	Incidence of grades 4-5 regimen-related toxicity	N/A	N/A
**CAR-T cells**								
Treatment of Relapsed and/or Refractory AQP4-IgG Seropositive NMOSD by Tandem CAR T Cells Targeting CD19 and CD20	NCT03605238	Withdrawn (recruitment failure)	Phase I	Tandem CAR-T cells against CD19 and CD20	Refractory NMOSD	Occurrence of study related adverse events	N/A	N/A
Safety and Efficacy of CT103A Cells for Relapsed/Refractory Antibody-associated Idiopathic Inflammatory Diseases of the Nervous System (CARTinNS)	NCT04561557	Recruiting	Dose-escalating phase I	CAR-T cells against BCMA, after lymphodepletion with cyclofosfamide and fludarabine	Refractory NMOSD	Dose-limiting toxicities and adverse events	N/A	N/A
**Mesenchymal stem cells**								
Autologous Mesenchymal Stem Cells for the Treatment of Neuromyelitis Optica Spectrum Disorders	NCT02249676	Completed	Placebo-controlled phase II	Autologous mesenchymal stem cells	Refractory NMOSD	EDSS change before and one year after infusion	EDSS reduction from 4.9 to 4.3	Fu et al. [70]
Safety and Efficacy of Umbilical Cord Mesenchymal Stem Cell Therapy for Patients With Progressive Multiple Sclerosis and Neuromyelitis Optica	NCT01364246	Completed	Phase I/II	Human umbilical cord mesenchymal stem cells	MS and AQP4+ NMOSD	EDSS	EDSS improvement with 6.5% ± 26.1% at 24 months following transplantation	Lu et al. [71]

## Data Availability

Data availability is not applicable to this article as no new data were created or analysed in this study.
